# Supplementary material for: Long-distance decay-less spin transport in indirect excitons in a van der Waals heterostructure
Source: Nat Commun. 2024 Nov 1;15:9454. doi: 10.1038/s41467-024-53445-5 (PMC11530635; doi:10.1038/s41467-024-53445-5)
Supplement: Supplementary file 1 — Supplementary Information [file 41467_2024_53445_MOESM1_ESM.pdf]

Supporting Information for
Long-distance decay-less spin transport in indirect excitons in a van der Waals heterostructure

Zhiwen Zhou,[1] E. A. Szwed,[1] D. J. Choksy,[1] L. H. Fowler-Gerace,[1] and L. V. Butov[1]
[1]*Department of Physics, University of California at San Diego, La Jolla, CA 92093, USA*

Contents

Supplementary Notes 1: The heterostructure details 2
Supplementary Notes 2: Optical measurements 3
Supplementary Notes 3: LE-IX and HE-IX spectral profile separation 3
Supplementary Notes 4: The density and temperature dependence of LE-IX transport 3
Supplementary Notes 5: The temperature dependence of co-polarized and cross-polarized IX PL 5
Supplementary Notes 6: Estimates of $d^{s}_{1/e}$ 6

References 8

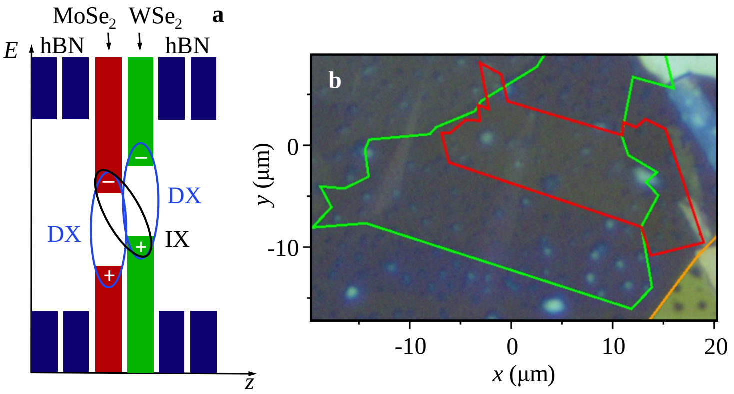

FIG. S1: (a) Schematic energy-band diagram for the $MoSe_2/WSe_2$ HS. The ovals indicate a direct exciton (DX) and an indirect exciton (IX) composed of an electron (–) and a hole (+). (b) A microscope image showing the layer pattern of the HS. The green and red lines indicate the boundaries of WSe_2 and $MoSe_2$ monolayers, respectively. The bottom and top hBN layers entirely cover the WSe_2 and $MoSe_2$ monolayers, the boundaries of bottom hBN layer are beyond the figure and a part of the boundaries of top hBN layer is shown by the orange line.

Supplementary Notes 1: The heterostructure details

The van der Waals $MoSe_2/WSe_2$ heterostructure (HS) was assembled using the dry-transfer peel-and-lift technique [1]. The same HS was used for the studies of IX transport in Ref. [2] and the HS manufacturing details are described in Ref. [2]. The thickness of bottom and top hBN layers is about 40 and 30 nm, respectively. The $MoSe_2$ monolayer is on top of the WSe_2 monolayer. The long WSe_2 and $MoSe_2$ edges reach ~ 30 and ~ 20 μm, respectively, which enables a rotational alignment between the WSe_2 and $MoSe_2$ monolayers. The twist angle $\delta\theta = 1.1^\circ$ corresponding to the moiré superlattice period $b = 17$ nm, which gives $N \sim 1/2$ at the estimated $n \sim 2 \times 10^{11}$ cm^{-2} for the long-distance IX transport as outlined in the main text, agrees with the angle between $MoSe_2$ and WSe_2 edges in the HS (Fig. S1b).

The accuracies of estimating $\delta\theta$ using the long WSe_2 and $MoSe_2$ edges and using SHG are comparable. We do not use SHG for additional estimates of $\delta\theta$ since the intense optical excitation pulses in SHG measurements may cause a deterioration of the HS and may suppress both the long-distance IX transport and the long-distance spin transport. As outlined in the main text, the moiré potentials can be affected by atomic reconstruction and by disorder and may vary over the HS area.

Figure S1b presents a microscope image showing the layer pattern of the HS. The layer boundaries are indicated. The hBN layers cover the entire areas of $MoSe_2$ and WSe_2 layers. There was a narrow multilayer graphene electrode on the top of the HS around $x = 2$ μm for $y = 0$, Fig. S1b. This electrode was detached. The IX luminescence reduction around $x = 2$ μm can be related to residual graphene layers on the HS.

We did not verify if stacking in the sample is R or H (AA or AB). This is the subject for future works. We note however, that regardless the stacking type, the data in the paper demonstrate the proof-of-principle for the existence of long-distance spin transport in TMD HS. We note also that the characteristic energies in the IX system in the regime of the long-distance spin transport (Fig. 3), including the IX interaction energy $\delta E \sim 3$ meV and thermal energy $k_B T \lesssim 1$ meV (k_B is the Boltzmann constant), are considerably smaller than the estimated amplitudes of the moiré potential for both R and H stacking [3–6].

So far, the long-distance spin transport in IXs in TMD HS was realized in one sample in this work. Other samples show spin transport in IXs with shorter decay distances [7–10]. The shorter range of spin transport likely originates from disorder, which causes scattering and, in turn, spin relaxation [11]. The data in this work demonstrate the proof-of-principle for the existence of the long-distance spin transport in IXs and the measured range of parameters for this phenomenon allows comparing it with the theory, as outlined in the main text. It is essential to study this phenomenon in other samples with different HS parameters. However, the sample statistics outlined above shows that it is challenging to manufacture samples with different HS parameters, all with sufficiently small disorder. It is also essential to study this phenomenon in other samples with larger sizes. However, the sizes of HS manufactured so far [7–10] are comparable to the sizes of HS studied in this work, indicating that increasing the HS sizes is challenging. This remains the subject for future works.

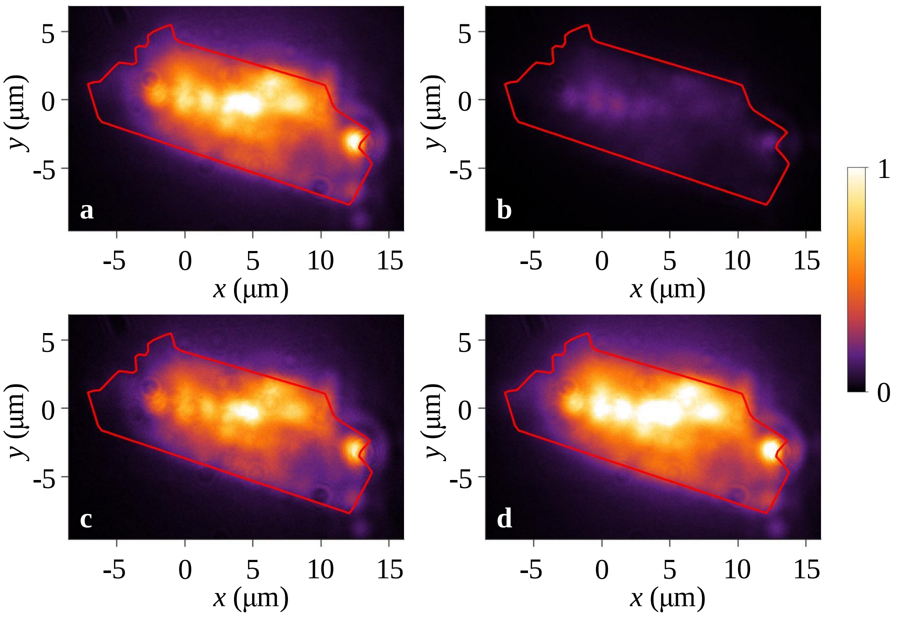

FIG. S2: (a,b) IX PL images co-polarized $I_{\sigma^+}(x,y)$ (a) and cross-polarized $I_{\sigma^-}(x,y)$ (b) with the circularly polarized laser excitation. The IX PL is selected by a filter $E \lesssim 1.4$ eV. (c) The spin density image $I_{\rm spin} = I_{\sigma^+} - I_{\sigma^-}$. (d) The density image $n = I_{\sigma^+} + I_{\sigma^-}$. The $\sim 2\ \mu$m laser excitation spot is centered at $(0,0)$, $P_{\rm ex} = 0.2$ mW, $T = 1.7$ K.

Supplementary Notes 2: Optical measurements

The continuous-wave polarization-resolved PL experiments are outlined in Section Methods in the main text. Figure S2 shows representative polarization-resolved IX PL images.

The IX PL kinetics in the sample was measured in earlier studies [2] using a pulsed semiconductor laser and a liquid-nitrogen-cooled CCD coupled to a PicoStar HR TauTec time-gated intensifier. These measurements showed that the IX lifetimes in the sample are $\sim 10 - 20$ ns for the densities and temperatures corresponding to both the long-distance IX transport and the long-distance spin transport carried by IXs.

These measurements also showed that for the densities and temperatures corresponding to both the long-distance IX transport and the long-distance spin transport carried by IXs, the IX propagation is characterized by the average velocity of the IX cloud expansion $v \sim \Delta R/\Delta t \sim 5 \times 10^4$ cm/s [2]. Since the spin transport is carried by IXs, the IX transport kinetics can give a rough estimate for the spin transport kinetics, however, spatially- and polarization-resolved imaging experiments are needed to measure the spin transport kinetics in IXs and this is the subject for future works.

Supplementary Notes 3: LE-IX and HE-IX spectral profile separation

We used two methods to separate LE-IX spectra from HE-IXs spectra: (i) by the spectral integration in the range $E < 1.4$ eV where LE-IXs dominate the spectra (Fig. 1a and 3a) and (ii) by the gaussian fits (Fig. S3b). These two methods give similar results: compare Fig. 1b,c with Fig. S4a,b, Fig. 1d-f with Fig. S5a-c, and Fig. 2a-c with Fig. S6a-c where the LE-IX spectra were separated from HE-IX spectra using these two different methods in the two figures in each pair of the figures.

Supplementary Notes 4: The density and temperature dependence of LE-IX transport

The LE-IX transport is characterized by the $1/e$ decay distance $d_{1/e}$ of the LE-IX PL intensity $I = I_{\sigma^+} + I_{\sigma^-}$ (Figs. S7 and S8). In these two figures LE-IX spectra are separated from HE-IX spectra using the two methods outlined above. Figures S7 and S8 show that these two methods give similar results.

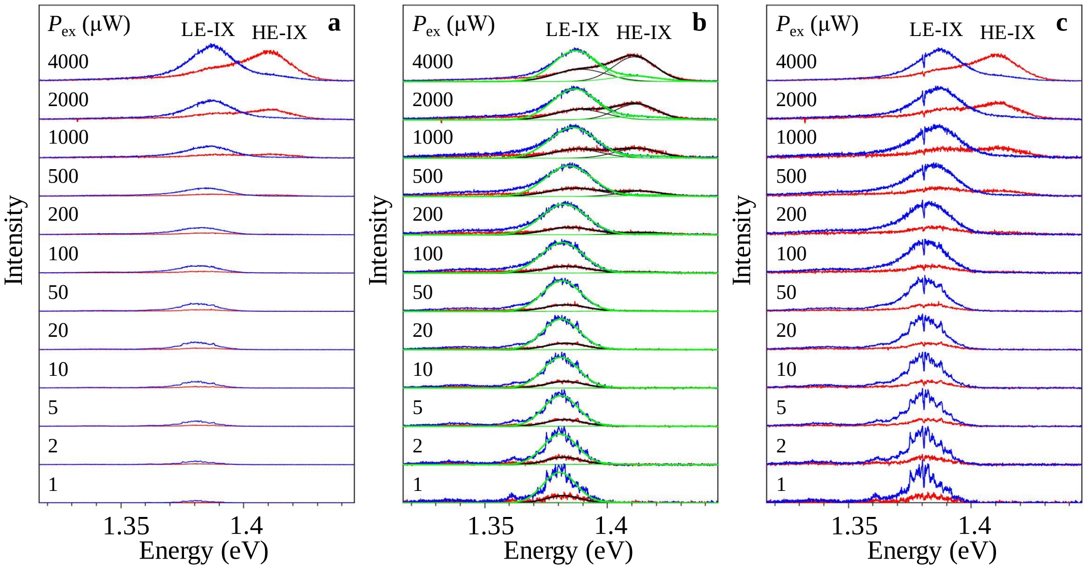

FIG. S3: (a) The excitation power dependence of co-polarized (blue) and cross-polarized (red) IX spectra. The LE-IX PL is co-polarized. The HE-IX PL is cross-polarized. The HE-IXs appear in the spectra at high $P_{ex} \gtrsim 0.2$ mW. The same spectra with normalized intensities are shown in Fig. 3a. (b) The same spectra as in Fig. 3a with the spectral profile separation of the co-polarized and cross-polarized PL of LE-IXs and HE-IXs. The gaussian fits to the co-polarized (cross-polarized) LE-IX spectra and HE-IX spectra are shown by the thin green (black) lines. The sum of the gaussians shown by the thin green (black) lines is shown by the thick green (black) line. (c) The same spectra as in (b) without excluding the part of the spectra affected by the CCD defect at ~ 1.38 eV, which causes an intensity reduction. The parts of the spectra affected by this defect are excluded from the spectra in (b) and other spectra in the paper. $T = 3.5$ K.

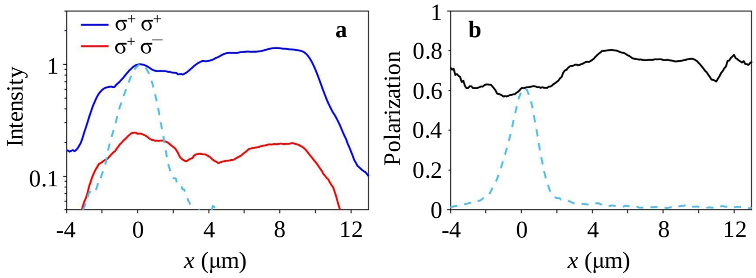

FIG. S4: Same as Fig. 1b,c for LE-IXs with the LE-IX spectra separated from the HE-IX spectra by the gaussian fits. Selecting LE-IXs by the spectral integration in the range $E < 1.4$ eV (as in Fig. 1b,c) or by the spectral integration of the gaussian fits (as in Fig. S4a,b) give similar results.

The density and temperature dependence of LE-IX transport (Figs. S7 and S8) is qualitatively similar to the density and temperature dependence of IX transport [2]. The latter refers to transport of all IXs, including LE-IXs and HE-IXs, and has a narrower $d_{1/e}(P_{ex})$ profile, in particular, because HE-IXs have shorter transport distances. The density and temperature dependence of spin transport in LE-IXs (Fig. 2) is qualitatively similar to the density and temperature dependence of LE-IX transport (Figs. S7 and S8) as outlined in the main text.

The LE-IX intensity enhancement with density slows at intermediate densities (Fig. 3b). This is qualitatively consistent with a more effective LE-IX cloud expansion from the excitation spot due to the longer-distance LE-IX transport at these densities (Figs. S7 and S8).

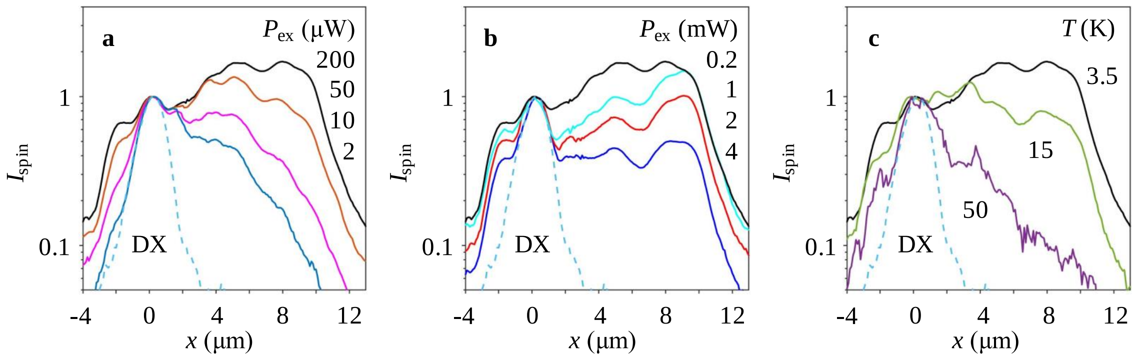

FIG. S5: Same as Fig. 1d-f for the LE-IXs with the LE-IX spectra separated from the HE-IX spectra by the gaussian fits. Selecting LE-IXs by the spectral integration in the range $E < 1.4$ eV (as in Fig. 1d-f) or by the spectral integration of the gaussian fits (as in Fig. S5) give similar results.

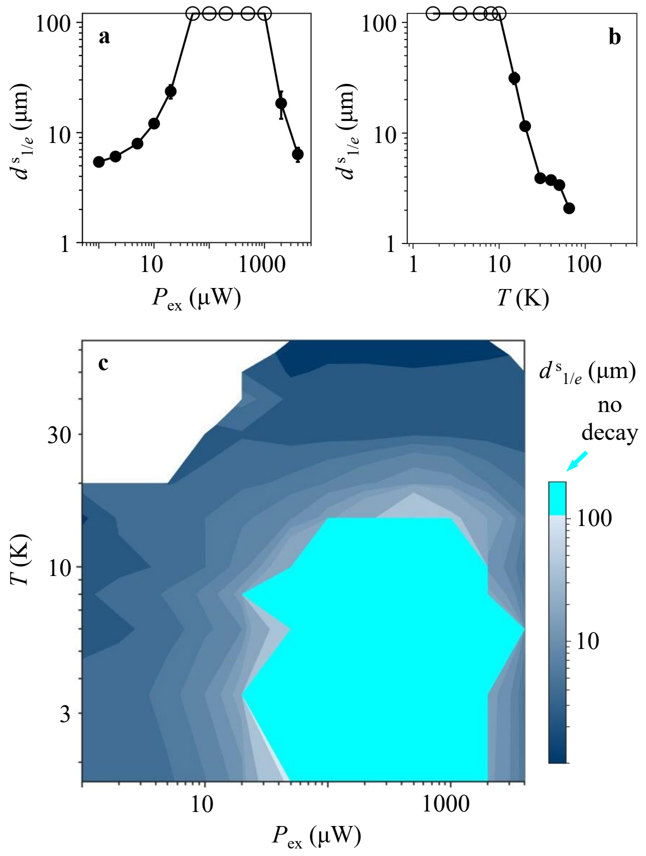

FIG. S6: Same as Fig. 2 for LE-IXs with the LE-IX spectra separated from the HE-IX spectra by the spectral integration in the range $E < 1.4$ eV. Selecting LE-IXs by the spectral integration in the range $E < 1.4$ eV (as in Fig. S6) or by the spectral integration of the gaussian fits (as in Fig. 2) give similar results.

Supplementary Notes 5: The temperature dependence of co-polarized and cross-polarized IX PL

The temperature dependence of co-polarized and cross-polarized IX spectra is presented in Fig. S9. The temperature increase causes a reduction of the degree of circular polarization of LE-IX PL $P = (I_{\sigma^+} - I_{\sigma^-})/(I_{\sigma^+} + I_{\sigma^-})$ (Fig. S9c). However, this reduction is rather small, with no sharp changes at $T \sim 10$ K where the transport of spin polarization

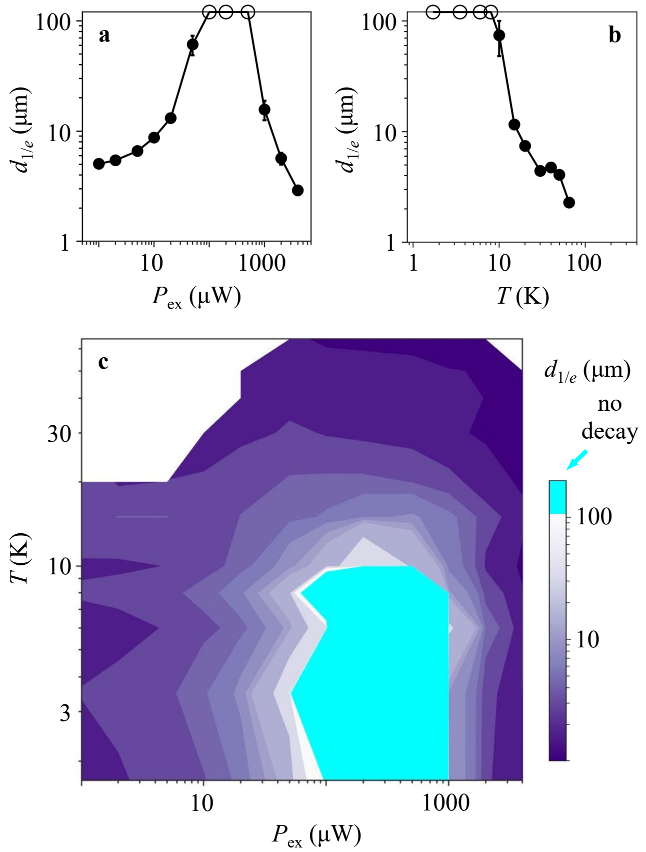

FIG. S7: Excitation power and temperature dependence of LE-IX transport. (a,b) The $1/e$ decay distance $d_{1/e}$ of LE-IX PL intensity $I = I_{\sigma^+} + I_{\sigma^-}$ vs. P_{ex} (a), vs. temperature (b), and vs. P_{ex} and temperature (c). $d_{1/e}$ are obtained from least-squares fitting the LE-IX intensity profiles $I(x)$ to exponential decays in the region from the excitation spot to the HS edge, $x = 0 - 9$ μm. The data with the fit indicating diverging $d_{1/e}$ are presented by circles on the edge (a,b) or by cyan color (c). The error bars represent the uncertainty in least-squares fitting the LE-IX transport decays to exponential decays. The LE-IX spectra are separated from the HE-IX spectra by the spectral integration of the gaussian fits. $T = 3.5$ K (a), $P_{ex} = 0.2$ mW (b).

density I_{spin} carried by LE-IX sharply drops (Fig. 2). This confirms that the dominant mechanism of the decay of spin density $I_{spin} = Pn$ is the decay of IX density.

Supplementary Notes 6: Estimates of $d^s_{1/e}$

The spin density transport $1/e$ decay distances $d^s_{1/e}$ (Fig. 2) are extracted from least-squares fitting the LE-IX spin density transport profiles $I_{spin}(x)$ (Fig. 1d-f) to exponential decays in the region from the excitation spot to the HS edge, $x = 0 - 9$ μm. There is no IX signal outside HS (Fig. 1b). For the fit of the data in the range $x = 0 - 9$ μm, the uncertainties in the fit allow determining $d^s_{1/e}$ up to ~ 100 μm: For $d^s_{1/e} \lesssim 100$ μm (Fig. 2a,b), the uncertainties in the fit, shown by the error bars when larger than the point size, are small; however, for the range of excitation densities and temperatures where the decay distances extracted from the fit become higher than ~ 100 μm, the uncertainties become large and comparable to the extracted decay distances. For instance, the fit gives $d^s_{1/e} = 90 \pm 30$ μm at $T = 15$ K and $P_{ex} = 0.5$ mW (Fig. S10). This example presents $d^s_{1/e}$ at the parameters where the value of $d^s_{1/e}$ determined from the fit is high yet finite. When approaching the temperatures and densities shown by open circles in Fig. 2a,b and by cyan color in Fig. 2c, the decay lengths in the fits become larger and diverge. The IX transport $1/e$ decay distances $d_{1/e}$ are similarly estimated.

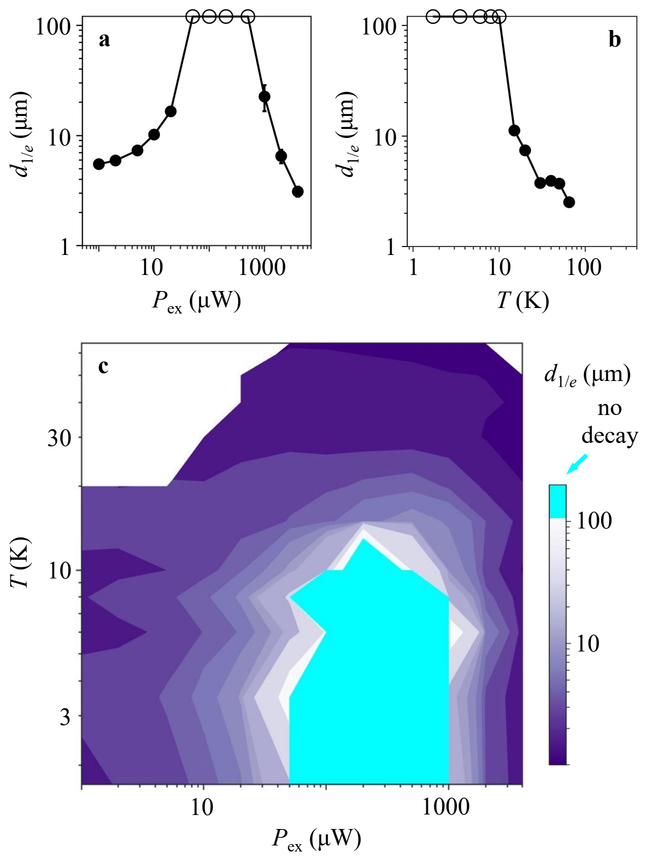

FIG. S8: Same as Fig. S7 for LE-IXs with the LE-IX spectra separated from the HE-IX spectra by the spectral integration in the range $E < 1.4$ eV. Selecting LE-IXs by the spectral integration in the range $E < 1.4$ eV (as in Fig. S8) or by the spectral integration of the gaussian fits (as in Fig. S7) give similar results.

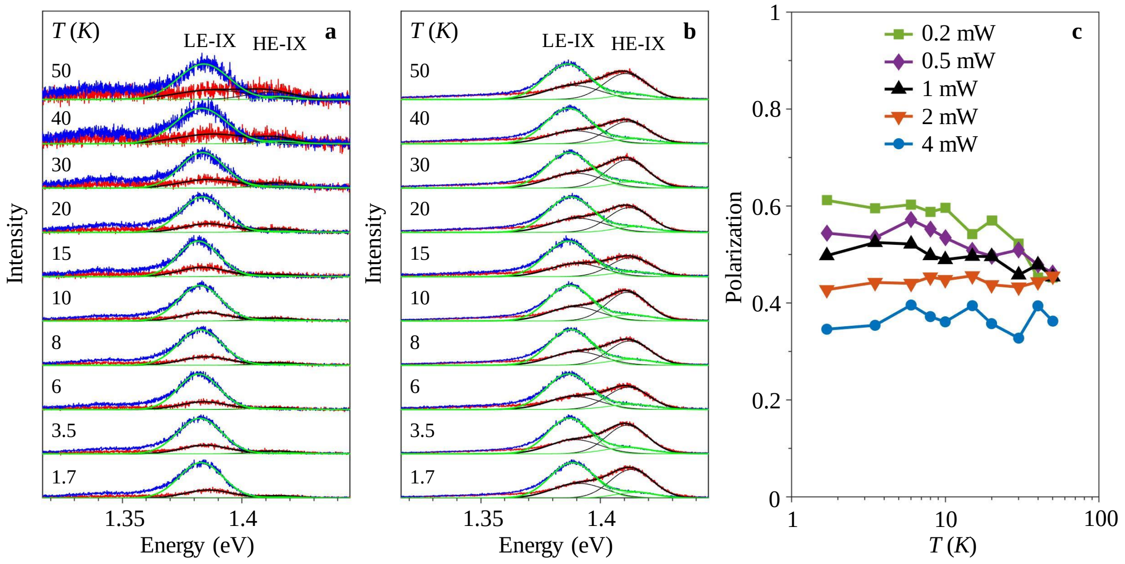

FIG. S9: (a,b) The temperature dependence of co-polarized (blue) and cross-polarized (red) IX spectra at $P_{ex} = 0.2$ mW (a) and 4 mW (b). The LE-IX PL is co-polarized. The HE-IX PL is cross-polarized. The HE-IXs appear in the spectra at high $P_{ex} \gtrsim 0.2$ mW. The intensities are normalized. (c) The degree of circular polarization of LE-IX PL $P = (I_{\sigma^+} - I_{\sigma^-})/(I_{\sigma^+} + I_{\sigma^-})$ vs. temperature at different P_{ex}. The LE-IX spectra are separated from the HE-IX spectra by the gaussian fits.

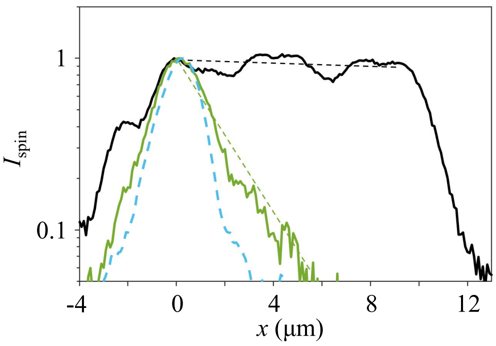

FIG. S10: Normalized spin density profiles $I_{spin} = I_{\sigma^+} - I_{\sigma^-}$ for the LE-IXs for the long-distance spin-valley transport (black line, T = 15 K and P_{ex} = 0.5 mW) and shorter-distance spin-valley transport (green line, T = 65 K and P_{ex} = 0.5 mW). The black and green dashed lines show decays with the $1/e$ decay distances 92 and 2 μm, respectively. The blue dashed line shows the DX luminescence profile in the $MoSe_2$ ML, this profile is close to the laser excitation profile for short-range DX transport. The ∼ 2 μm laser spot is centered at $x = 0$.

[1] F. Withers, O. Del Pozo-Zamudio, A. Mishchenko, A.P. Rooney, A. Gholinia, K. Watanabe, T. Taniguchi, S.J. Haigh, A.K. Geim, A.I. Tartakovskii, K.S. Novoselov. Light-emitting diodes by band-structure engineering in van der Waals heterostructures. *Nat. Mater.* **14**, 301 (2015).

[2] L.H. Fowler-Gerace, Zhiwen Zhou, E.A. Szwed, D.J. Choksy, L.V. Butov. Transport and localization of indirect excitons in a van der Waals heterostructure. *Nat. Photonics* **18**, 823 (2024).

[3] Fengcheng Wu, Timothy Lovorn, A.H. MacDonald. Theory of optical absorption by interlayer excitons in transition metal dichalcogenide heterobilayers. *Phys. Rev. B* **97**, 035306 (2018).

[4] Hongyi Yu, Gui-Bin Liu, Wang Yao. Brightened spin-triplet interlayer excitons and optical selection rules in van der Waals heterobilayers. *2D Mater.* **5**, 035021 (2018).

[5] Fengcheng Wu, Timothy Lovorn, A.H. MacDonald. Topological Exciton Bands in Moiré Heterojunctions. *Phys. Rev. Lett.* **118**, 147401 (2017).

[6] Hongyi Yu, Gui-Bin Liu, Jianju Tang, Xiaodong Xu, Wang Yao. Moiré excitons: From programmable quantum emitter arrays to spin-orbit–coupled artificial lattices. *Sci. Adv.* **3**, e1701696 (2017).

[7] Pasqual Rivera, Kyle L. Seyler, Hongyi Yu, John R. Schaibley, Jiaqiang Yan, David G. Mandrus, Wang Yao, Xiaodong Xu. Valley-polarized exciton dynamics in a 2D semiconductor heterostructure. *Science* **351**, 688 (2016).

[8] Dmitrii Unuchek, Alberto Ciarrocchi, Ahmet Avsar, Zhe Sun, Kenji Watanabe, Takashi Taniguchi, Andras Kis. Valley-polarized exciton currents in a van der Waals heterostructure. *Nat. Nanotechnol.* **14**, 1104 (2019).

[9] Zumeng Huang, Yuanda Liu, Kévin Dini, Qinghai Tan, Zhuojun Liu, Hanlin Fang, Jin Liu, Timothy Liew, Weibo Gao. Robust Room Temperature Valley Hall Effect of Interlayer Excitons. *Nano Lett.* **20**, 1345 (2020).

[10] Daniel N. Shanks, Fateme Mahdikhanysarvejahany, Trevor G. Stanfill, Michael R. Koehler, David G. Mandrus, Takashi Taniguchi, Kenji Watanabe, Brian J. LeRoy, John R. Schaibley. Interlayer Exciton Diode and Transistor. *Nano Lett.* **22**, 6599 (2022).

[11] M.I. Dyakonov. *Spin Physics in Semiconductors* (Springer, New York, 2008).
